# A systematic review and narrative analysis of digital speech biomarkers in Motor Neuron Disease

**DOI:** 10.1038/s41746-023-00959-9

**Published:** 2023-12-07

**Authors:** Molly Bowden, Emily Beswick, Johnny Tam, David Perry, Alice Smith, Judy Newton, Siddharthan Chandran, Oliver Watts, Suvankar Pal

**Affiliations:** 1 College of Medicine and Veterinary Medicine, University of Edinburgh, Edinburgh, UK; 2 Anne Rowling Regenerative Neurology Clinic, University of Edinburgh, Edinburgh, UK; 3 Centre for Clinical Brain Sciences, University of Edinburgh, Edinburgh, UK; 4 Euan MacDonald Centre for Motor Neuron Disease, University of Edinburgh, Edinburgh, UK; 5 SpeakUnique, Edinburgh, UK; 6 UK Dementia Research Institute, London, UK

**Keywords:** Amyotrophic lateral sclerosis, Prognostic markers, Diagnostic markers

## Abstract

Motor Neuron Disease (MND) is a progressive and largely fatal neurodegeneritve disorder with a lifetime risk of approximately 1 in 300. At diagnosis, up to 25% of people with MND (pwMND) exhibit bulbar dysfunction. Currently, pwMND are assessed using clinical examination and diagnostic tools including the ALS Functional Rating Scale Revised (ALS-FRS(R)), a clinician-administered questionnaire with a single item on speech intelligibility. Here we report on the use of digital technologies to assess speech features as a marker of disease diagnosis and progression in pwMND. Google Scholar, PubMed, Medline and EMBASE were systematically searched. 40 studies were evaluated including 3670 participants; 1878 with a diagnosis of MND. 24 studies used microphones, 5 used smartphones, 6 used apps, 2 used tape recorders and 1 used the Multi-Dimensional Voice Programme (MDVP) to record speech samples. Data extraction and analysis methods varied but included traditional statistical analysis, CSpeech, MATLAB and machine learning (ML) algorithms. Speech features assessed also varied and included jitter, shimmer, fundamental frequency, intelligible speaking rate, pause duration and syllable repetition. Findings from this systematic review indicate that digital speech biomarkers can distinguish pwMND from healthy controls and can help identify bulbar involvement in pwMND. Preliminary evidence suggests digitally assessed acoustic features can identify more nuanced changes in those affected by voice dysfunction. No one digital speech biomarker alone is consistently able to diagnose or prognosticate MND. Further longitudinal studies involving larger samples are required to validate the use of these technologies as diagnostic tools or prognostic biomarkers.

## Introduction

Motor neuron disease (MND) is a devastating, progressive and largely fatal neurodegenerative disorder with characteristic upper and lower motor neuron pathology leading to deterioration in mobility, respiratory failure, and bulbar dysfunction with associated impaired communication^1,2^. MND is characterised by heterogeneity in presentation and disease progression. Individuals with the commonest subtype, amyotrophic lateral sclerosis (ALS) (affecting 70%) present with limb weakness, muscle wasting and often spasticity. Bulbar-onset ALS is characterised by prominent speech dysfunction as a presenting symptom. However, speech and language dysfunction affect the majority as disease progresses^1^. Up to 85% of pwMND experience bulbar dysfunction during their disease, regardless of the subtype^2–4^. Speech disturbance and communication difficulties have a significant impact on quality of life for pwMND.

Dysarthria is a speech disorder arising due to neurological impairment of the motor components of speech production. It is commonly the presenting symptom in bulbar-onset ALS. The aetiology of dysarthria in MND is multifactorial. Progressive dysfunction and weakness of motor neurons results in a global loss of bulbar muscle control and a reduction in speech functioning. Dysarthria is also commonly accompanied by swallowing difficulties and saliva problems involving abnormal saliva production and management^5–8^. The processing and production of language is also affected in pwMND. MND presents in a continuum with frontotemporal dementia (FTD). Up to 15% of pwMND fulfil cognitive and behaviour impairment satisfying a diagnosis of FTD and up to 50% display signs of impairment in cognitive and language domains, including expressive speech difficulties, and incorrect speech content^9^.

Currently, most individuals with MND are diagnosed based on a constellation of clinical features and supportive findings on electromyography (EMG). Inter-disciplinary clinical management of disease progression is essential^10^ and includes longitudinal evaluation of change with validated rating scales and respiratory function tests. Speech and language therapists and speech-language pathologists are instrumental in the assessment and management of speech difficulties^11^. Current speech assessments in clinical practice are mainly qualitative and subjective, and there are low levels of standardisation and comparability between assessors.

Assessment of bulbar involvement is complex and encompasses a wide range of clinical assessments and diagnostic tools. Recent studies have reviewed emerging methods in the assessment of dysarthria and dysphagia in MND^12^, and acoustic assessment in bulbar-ALS^13^. Most routinely conducted clinical assessments remain qualitative and subjective, although more standardised assessment tools such as the Frenchay Dysarthria Assessment are available^14^. The absence of standardised protocols and reliance on specialised equipment pose challenges to the assessment process. Perceptual rating scales administered by healthcare professionals are more commonly used and involve expert evaluation of features such as speech intelligibility, articulation clarity, voice quality and prosody. Clinician rated perceptual evaluation tools currently offer more accessible and practical assessment methods. One frequently used measure of ALS progression is the ALS Functional Rating Scale-Revised (ALS-FRS(R)). Speech intelligibility is included as only a single item rated on a scale of 0 to 4 (4 representing normal) in this validated 12 item, 48 point clinician-administered questionnaire; although the scale has been reported to lack objectivity^15^. More detailed approaches include laryngeal function evaluation and kinematic jaw function analysis^7,16^ although these methods are only used infrequently outside of research settings.

Digital technologies for speech data acquisition and analysis have potential to offer a more reliable, objective, and sensitive measure of deterioration than perceptual analysis and validated rating scales alone and may be useful as prognostic and diagnostic tools in MND. New technologies are increasingly being reported for diagnosis and management of MND with a focus on earlier identification and disease monitoring^17^.

In speech and language therapy and pathology, speech and voice are often distinguished as separate physiological entities. Voice is defined as the sound created by the vocal tract, whereas speech is defined as the articulatory movements that produce sounds of vowels and consonants. From the perspective of digital speech processing the two mechanisms constitute the same continuous process, so no distinction is usually made between the two. Instead, categorisation of speech features is usually based on the type of digital signal measures that can be obtained. These digital speech biomarkers can broadly be categorised into acoustic and linguistic features of speech^18^. Acoustic features are measures of the acoustic characteristics of produced speech derived using digital speech processing methods. Examples include measures of vocal quality such as jitter, shimmer, and harmonic-to-noise ratio (HNR), and spectral features such as formant trajectories. Linguistic features on the other hand are language-dependent textual information pertaining to the language content of produced speech, such as lexical, syntactical, and semantic features. These features usually require manual transcription or automated speech recognition algorithms. Characteristics of ‘paralanguage’ can also be extracted from speech, sometimes defined as the non-phonemic properties of speech such as respiration, prosody, pitch, and volume. These characteristics are occasionally included within acoustic features or defined separately as “paralinguistic features”.

The use of speech as a biomarker for diagnostic and prognostic evaluation in MND is rapidly evolving. Speech is attractive as a biomarker due to the potential for non-invasive, cost-effective, remote, and scalable (for example via apps), acquisition and analysis. However, considerable heterogeneity exists in study aims, speech sampling methods and speech features assessed.

### Aims and objectives

This systematic review aims to synthesise current evidence regarding digital speech biomarkers used for the diagnosis and monitoring of MND. This is achieved by the following specified obejectives:

1. Explore the use of digital speech assessment devices used in pwMND.
2. Identify the speech tasks used to elicit speech for digital speech assessment in MND.
3. Identify unique features of speech identified through digital speech assessment in patients with a diagnosis of MND.
4. Evaluate the clinical utility of digital speech biomarkers for use in MND diagnosis and progression monitoring.

For this systematic review we set the following hypotheses to be tested:

1. Studies will likely be exploratory with small sample sizes and duration of follow up, with a variation in the assessment technology used and a lack of consistency in study procedures or speech tasks.
2. Clinical correlation, if conducted, will be based on existing rating scales like the ALS-FRS(R).
3. Due to the exploratory nature of many of the studies, and the anticipated variability in the data, the current clinical utility of the investigated biomarkers will be limited.

## Results

**Fig. 1:**
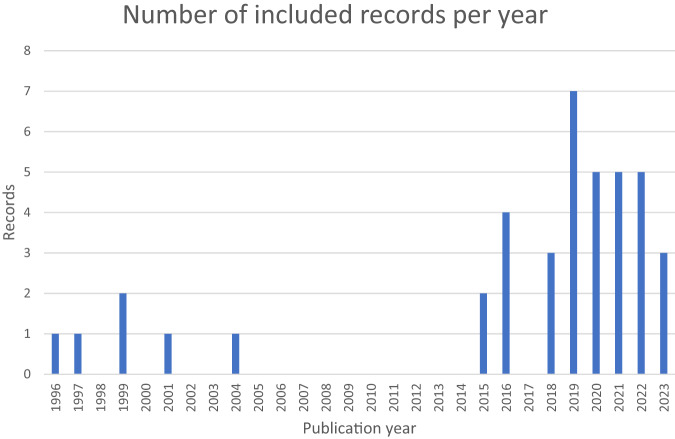
Number of relevant studies per year (1996–2023).

Initial searches yielded 1774 studies. Subsequently, 1405 titles and abstracts were screened with 94 full texts eligible for screening. Most studies identified were published in the last 5 years (Fig. 1).

40 studies were included in the final analysis, summarised in the PRISMA diagram in Fig. 3. The 40 studies included a total of 3,670 participants and 1878 (51%) with a diagnosis of MND. Information regarding site of onset was available for 20 studies (50%). This included 300 participants with spinal-onset, 387 with bulbar-onset, and 261 with other. Duration of disease ranged from 5-57 months^19^. Non-MND participants included a total of 935 controls, 107 participants with a diagnosis of Parkinson’s disease (PD) and 148 with other neurological conditions including Kennedy’s Disease and FTD.

26 studies conducted assessments at a single time-point. The remaining 14 studies were longitudinal with follow-up periods of up to 60 months^16^. Data collection was performed at clinic sites in 32 (80%) studies, exclusively at home in 6 (15%), and in a hybrid model of clinic and home settings for a single study. One study analysed speech samples from two separate databases, one which carried out in-clinic assessment and another using remote assessment^20^. Participant experience was recorded in 3 (8%) studies^21–23^, with adverse effects reported in 2 of these^22,23^.

Each objective is addressed sequentially. An overview of the studies included is provided in Table 1. A supplementary table details digital speech assessments devices (Supplementary Table 1) and speech tasks and speech features (Supplementary Table 2).

**Table 1:** Overview of included studies.

First Author and Publication Year	Number of participants with ALS (Number with Bulbar Onset)	Length of follow up	Frequency of data collection	Speech sample acquisition method	Device Brand	Speech features assessed	Additional assessments	Mean Total ALS-FRS (R) (Mean Bulbar Sub-score)	Results/Conclusions
Agurto, 2019^28^	42 (34)	11 months	Once weekly (ALS-FRS/FVC/SVC at start point)	App	Help us Answer ALS	Pitch variation, prosody features, vowel space, vowel quality and noise measurements, mel frequency cepstral coeffients (MFCC), tremor features including tremor frequency and tremor amplitude, spectral features (including spectral slope, the maximum energy, the frequency where the maximum value is obtained, as well as the median and the energy IQR of the long-term average spectra)	ALS-FRS(R) FVC SVC	F-34.70 M-40.50 (-)	AR and MFCC highly correlated with initial speech score. Best speech features obtained from the reading task. Initial scores unable to determine disease trajectory but extracted features could. Progression related more to onset type than initial score.
Berry, 2019^27^	23 (4)	24 weeks	Twice weekly for passage reading. 4 days per week for 3 randomly assigned questions ALSFRS-(R) in full once weekly 6, 12 and 24 week clinician administered ALS-FRS(R)	App	Beiwe	Speech and pause variables	ALS-FRS(R) VC ALS-CBS	34.00 (-)	The correlation between clinic assessed and app ALS-FRS(R) scores taken at a single time point was high at 0.93. High correlation for trajectory of decline between clinic assessment and app-based assessment. An increase in pause duration was demonstrated over time.
Buder, 1996^31^	1 (1)	2 years	3 assessments completed	Audiocasset	-	FORMOFFA (FOR= FORmants; MO =MOments; FF = Fundamental Frequency; A = Amplitude).	None	-	Reduction in amplitude (flattening) and reduced spectral mean variability (M1).
Cebola, 2023^53^	40 (-)	Single-time point	-	Smartphone	-	Window-based features (temporal, spectral and statistical). Full signal features (silence features and formant features).	None	-	Sample based classification: Best overall data was from the vowel task. Best results came from use of the whole feature set. The best results were achieved with SVM for both datasets. Patient based classification: Achieved better results than sample-based with the best results achieved using the complete feature set. Accuracy and F1 scores also improved. Accuracy values of over 0.90 using the full speech feature set.
Chiaramonte, 2019^10^	22 (22)	3 months	Monthly	Multi-Dimensional Voice Programme (MDVP)	Multi-Dimensional Voice Programme (MDVP)	F0; long-term control of amplitude (vAM); jitter; shimmer; Noise to harmonic ratio (NHR); Amplitude tremor intensity index (ATRI); Frequency tremor intensity index (FTRI); Voice turbulence index (VTI); Soft phonation index (SPI); Degree of subharmonic components (DSH) and Degree of voice break (DVB).	GIRBAS Scale Penetration Aspiration Scale ENT assessment Spectrogram Electroglottography FEES	-	Jitter, shimmer, vF0, ATRI, FTRI and vAM all significantly increased. NHR significantly reduced. The scores of the GIRBAS scale and Penetration Aspiration Scale (PAS) were higher at 3 months than at initial assessment, indicating increased difficulty in swallowing. Both progressive dysphagia and dysarthria are associated with muscle weakness and loss of motor control. Acoustic analysis should be used in combination with other assessments including swallowing assessment.
Garcia-Gancedo, 2019^22^	25 (2)	48 weeks	Clinic visit every 12 weeks (speech assessment) Wore sensor for 3 consecutive days every month	Microphone	High fidelity speech capture system	Central tendency of F0, jitter, shimmer, speaking rate, average phoneme rate and % pause time	ALS-FRS(R) FVC	41.60 (-)	All (100%) patients captured the digital speech data successfully at baseline and at week 48. All collected data was successfully analysed. Little change from baseline or an observable change over time was seen. Recording of voice samples using this method is feasible and acceptable to patients.
Kelly, 2020^23^	25 (2)	48 weeks	Clinic visit every 12 weeks (speech assessment) Wore sensor for 3 consecutive days every month	Microphone	High fidelity speech capture system	Central tendency of F0, jitter, shimmer, speaking rate, average phoneme rate and % pause time	ALS-FRS(R) FVC	41.60 (11.40)	Four speech endpoints showed between-patient correlation coefficient >0.5- with bulbar (average phoneme rate and average speaking rate) and respiratory (jitter and shimmer) of ALS-FRS(R) score.
Laganaro, 2021^32^	20 (-)	Single-time point	-	Microphone	-	Intelligibility, articulation, pneumophonatory control. Voice features: jitter; shimmer; F0, harmonic to noise ratio (HNR), cepstral peak prominence (CPPs); prosody, speaking rate and diadochokinetic rate (DDK).	None	-	High correlation between device system score and externally validated perceptual score. Sensitivity of 83.3% and specificity 95.2%. System able to identify abnormal speech but not distinguish between pathologies.
Lévêque, 2022^33^	33 (33)	Single-time point	-	Earset Microphone	Focusrite Scarlett (2i4) external audiocard and a professional quality Shure SM35-XLR earset microphone	TSC_MFCCs: Degree of acoustic change across a sequence. Mean TSC_MFCCs: Average acoustic change across a sequence. VARCO: Degree of variability of the acoustic change across a sequence. eventDUR: Differences in articulation rate.	None	-	MEAN acoustic change was significantly smaller for ALS and primary lateral sclerosis (PLS) compared with spinal and bulbar muscular atrophy SBMA (respectively *p* < .001 and *p* < .01) and controls (*p* < .001). ALS showed higher VARCO values than control (*p* < .0001), PLS (*p* = .04), and SBMA (*p* = .001). ALS and PLS speakers are slower than both SBMA and control speakers for all sequences.
Likhachov, 2021^30^	31 (-)	Single-time point	-	Smartphone	ALS Expert Mobile Application for Android.	Jitter. Shimmer. Features based on F0-contour: Pitch period entropy (PPE) and Pathological vibrato index (PVI). Noise features: Harmonic to noise ratio (HNR) and Glottal -to noise excitation ratio (GNE).	None	-	Classifier using jitter, PPE, PVI and GNE was the most accurate. Correct identification of ALS possible using only one voice test.
Liscombe, 2021^35^	50 (-)	Single-time point	-	Microphone	-	Speech events and silence events measured as: true negative time, true positive time, false negative and false positive. Speaker loudness.	ALS-FRS(R)	-	More silences present for the bulbar cohort most extremely demonstrated by the SIT task. Optimal configuration differs between groups.
Liscombe, 2023^34^	10 (-)	Single-time point	-	Microphone	-	Speech events and silence events measured as: true negative time, true positive time, false negative and false positive. Speaker loudness.	ALS-FRS(R)	-	Most dramatic change between control VAD settings and pathological VAD settings was endSilence which doubled. When VAD settings were optimised for pathological groups compared to control settings, DCF, I% and FN% reduced. These fell further when settings were further optimised for data in a specific cohort. Cohort specific 10-fold cross validation tests to assess robustness found little variation from other data presented.
Maffei, 2023^36^	49 (7)	Single-time point	-	Lapel Microphone	Audio-Technica AT831R	Perturbation and Noise based measures: local jitter; local shimmer; harmonic to noise ratio (HNR). Cepstral/spectral measures: cepstral peak prominence (CPP); Low High Spectral Ratio and Cepstral Spectral Index of Dysphonia (CSID)	None	-	Jitter, shimmer, and HNR levels are abnormal in ALS and can discriminate between normal and dysphonic voices. Cepstral/spectral measures also discriminated the groups with excellent or acceptable diagnostic accuracy, defined as an area under the curve (AUC) > .8 and > .7.
Mori, 2004^37^	4 (-)	Single-time point	-	Microphone	Dynamic microphone or electret condenser microphone MI-1233	F0 range and F0 minimum. F1: First formant frequency. F2: Second formant frequency	None	-	F0 range narrower in dysarthric speakers. F0 minimum for ALS did not differ significantly from controls. F1 and F2 vowel spaces were narrower than controls. Formant frequencies in expected regions.
Naeini, 2022^38^	243 (-)	Single time-point	-	Microphone	-	Pause duration; total duration; speech duration; pause events; % pause; mean phrase; coefficient of variation of phrase durations; coefficient of variation of pause durations	SIT	-	Both MFA and Wav2Vec2 performed well when compared to the Speech and Pause Analysis (SPA) software. Wav2Vec2 generalized better across clinical severities. Wav2Vec2 model performed better with most features. Audio deemed to be ‘good’ had the strongest correlations.
Neumann, 2021^51^	54 (32)	Single-time point	-	Microphone	-	Mean F0; jitter; shimmer; harmonic to noise ratio (HNR); cepstral peak prominence (CPP); speaking & articulation duration and rate; percentage pause time (PPT) and, Cycle-to-cycle temporal variation (cTV)	ALS-FRS(R)	Bulbar-33.09 (8.75) pre-bulbar-36. 45 (12.00)	Strong differences between acoustic features for timing measures. Effect sizes between bulbar and control groups were highest. Mean F0 showed a significant difference (smaller effect sizes). UAR- between control and pre-bulbar 0.63 and between bulbar and pre-bulbar -0.77. Voice quality measures added power to the predictive model for pre-bulbar samples.
Nevler, 2020^24^	67 (16)	Single-time point	-	-	Speech Activity Detector (SAD), developed at the University of Pennsylvania Linguistic Data Consortium.	F0, F0 range, mean speech segment duration, total speech duration, pause rate	Edinburgh Cognitive Assessment Scale (ECAS), ALS-FRS(R), Motor examination, Mini-Mental State Examination (MMSE) MRI	ALS- 35(-), ALS-FTD- 34.2 (-)	The F0 range was restricted in patients with ALS-FTD compared to healthy controls (*p* = 0.005). There was no significant difference between F0 range between motor ALS and healthy controls (p = 0.15). Regression analysis showed strong association between F0 range and severity of bulbar impairment. No association was found between F0 range and cognitive impairment using MMSE score as predictor of F0 range (*p* = 0.34). Mean speech segment duration was reduced in ALS-FTD compared to controls (*p* < 0.001) and motor-ALS (*p* = 0.042). Cognitive impairment was associated with mean speech segment duration and total speech time. Pause rate is related to cognitive function. Exploratory regression analysis revealed a relationship between F0 range, pause rate and total speech duration, and cortical thickness in different areas of the brain.
Norel, 2018^29^	67 (-)	Single-time point	-	App	ALS Mobile Analyzer	Mel-frequency cepstral coefficients (MFCC), spectral changes	ALS-FRS(R)	-	79% accuracy for males and 83% for females.For males a single feature could distinguish controls and patients. Model was tolerant to uncontrolled recording conditions.
Peplinski, 2019^25^	65 (-)	Several months	Daily	App	ALS at Home	Components of tremor: dominant tremor frequency, maximum absolute tremor intensity, median absolute tremor intensity, mean absolute tremor intensity, max relative tremor intensity, median relative tremor intensity, mean relative tremor intensity, tremor energy, tremor entropy	None	-	Discriminative power to separate perceptually rated tremor vs non-tremor. Unable to distinguish controls from those ALS without tremor.
Robert, 1999^39^	63 (40)	Single-time point	-	Digital tape recorder	-	F0, jitter, intensity, shimmer, number of harmonics in frequency spectral analysis.	None	-	5 of 8 acoustic features used present in symptomatic and asymptomatic ALS Jitter significantly higher with bulbar symptoms. Shimmer and CVF were also higher. No of harmonics was significantly lower in symptomatic ALS. MPFR was significantly lower in ALS patients
Rong, 2015^16^	66 (15)	60 months	Aimed every 3 months but varied based on clinic follow-up schedule. Average no of sessions was 7.	Microphone	Countryman E6 microphone	Respiratory subsystem: Pausing patterns and subglottal pressure. Phonatory subsystem: Jitter; shimmer; noise to harmonic ratio (NHR); loudness; and maximum F0. Resonatory subsystem: Nasalance, peak oral pressure and peak nasal airflow. Articulatory measures.	ALS-FRS(R) SIT	38.00 (10.00)	DDK and F0 identified early bulbar decline occurring before SR and SIT decline
Rong, 2016^40^	66 (15)	1792 days	Approximately every 3 months (varied with clinic schedule)	Microphone	Countryman E6	58 measures across 4 subsystems. Speech	ALS-FRS(R) %FVC SIT	38.00 (10.00)	Decline in AMR task performance prior to speech intelligibility decline. Distinction between fast and slow bulbar progressors.
Rong, 2020^19^	16 (-)	Single-time point	-	Microphone	-	Cycle-to-cycle temporal variation (cTV) and syllable rate (sylRate).	SIT	-	Cycle-to-cycle temporal variation (cTV) showed large increase in early bulbar disease. Large effect size of cTV between controls and early bulbar disease.
Rowe, 2022^20^	46 (-)	Single-time point	-	Microphone or App (depending on database used)	Professional quality microphones (e.g., AKG C410, Shure SM81 Condenser, Olympus VN-702PC digital recorder) or the Beiwe application	Coordination- relative duration of the silence between two articulatory gestures during each syllable transition (GapSyllProp). Consistency- across repetition variability in voice onset time. (RepVarVOT)Speed- Second formant slope in the consonant transition of /k/ (F2Slope). Precision - across-consonant variability in second formant slope in the consonant transitions of /p/, /t/, and /k/ (ConVarF2Slope). Rate - number of syllables produced per second (RepRate).	None	-	Multivariate analysis indicated a different articulatory pattern depending on the diagnosis of the speaker. This was significant for all articulatory components (coordination, speed, precision, rate). Overall Pearson correlation revealed only weak to moderate correlations with pairs of acoustic features for both each individual pathology and the whole study population. Speed and Precision were most strongly correlated (0.72) in speakers with ALS. ALS was the only clinical group where multivariate LDAs using receiver-operating characteristic (ROC) curves showed below acceptable values for sensitivity, specificity, and area under the curve (AUC). The full feature profile performed significantly better than the individual features at classifying the clinical groups.
Rutkove, 2020^21^	113 (60)	9 months	Daily for 90 days then 2x weekly for additional 180 days (ALSFRS(R) collected weekly)	App	ALS at home	-	ALS-FRS(R) FVC	36.10 (-)	Patients reported greater sense of control. Frequent at home data collection successful and would reduce future sample sizes.
Silbergleit, 1997^41^	20 (-)	Single-time point	-	Headband microphone	Cspeech CompuAdd computer, model 320/325 IBM ACPA (audio capture and playback adapter) A/D D/A card	Jitter; shimmer; Signal-to-noise ratio (SNR) and Maximum phonation frequency range (MPFR).	Hearing screening	-	Jitter and maximum phonation frequency range (MPFR) showed significant differences between groups. Shimmer and signal-to-noise ratio (SNR) unable to separate groups.
Stegmann, 2020^26^	65 (12)	9 months	Daily speech samples for 3 months then 2x weekly for 6 months (Average every 2.9 days)	App	ALS at home	AP & SR Articulatory precision (AP) and speaking rate (SR)	ALS-FRS(R)	37.10 (9.70)	Speaking rate (SR) and articulatory precision (AP) able to detect bulbar involvement early and track progression. Remote assessment via mobile app possible. Decline of AP and SR faster in bulbar-onset than non-bulbar onset.
Tanchip, 2022^42^	145 (33)	Single-time point	-	Microphone	Marantz PMD660 compact flash recorder with an accompanying Countryman E6 omnidirectional microphone or an Olympus WS-853 recorder with an accompanying ME52W unidirectional microphone	Diadochokinetic rate (DDK); cycle-to-cycle temporal variation (cTV); number of syllables	SIT	-	The intraclass correlation coefficient (ICC) calculated between syllable counts was 0.99 between both Raters 1 and 2 and Raters 2 and 3, suggesting excellent reliability of the manual procedure. Generally, there was overall agreement between the manual and algorithmic syllable detection. Disease severity had a significant effect on syllable count agreement (*p* < 0.001) with all five algorithms overestimating syllable count in the severe stage, and all except the Energy algorithm overestimating in the moderate stage. For DDK rate and cTV, the Energy algorithm performed best with correlations of over 0.7 with manual analysis.
Tena, 2022^43^	47 (14)	Single-time point	-	Microphone	USB EMITA Streaming GXT 252 microphone and Audacity (open-source application).	Phonatory subsystem features including: absolute jitter; relative jitter; absolute Shimmer; relative Shimmer; mean harmonic-to-noise ratio; pitch (SD), pitch (min), pitch (max), pitch (mean). Time frequency features including: Average instantaneous spectral energy, instantaneous frequency peak and spectral information	None	-	Differentiation of diagnosis by gender was the most important finding. The best model was Random Forest (RF). RF able to distinguish between control group and bulbar ALS patients with an accuracy of 96.1% and 98.1% for males and females respectively. Different numbers of statistically significant features were identified depending on the cohort and whether participants were male or female.
Tomik, 2015^44^	17 (17)	12 months	Baseline, at 6 months and 12 months	Microphone	-	F0, jitter, shimmer, noise-to-harmonic ratio (NHR), voice range and maximum phonation time (MPT).	None	-	Jitter was significantly higher for all examinations in women with ALS compared to controls. Mean shimmer and NHR values were significantly higher in women with ALS. Mean F0 did not show a reduction in ALS for either sex.
Tomik, 1999^45^	53 (15)	36 weeks	Every 10-12 weeks	Microphone	Bruel and Kjaer microphone	Articulation time, pause duration.	None	-	Significant differences between the mean distances for all chosen sounds in both ALS groups. Significant increase over time for mean distances for all sounds in both groups. Different acoustic signature patterns identified for each ALS group with different sounds showing different distance increases.
Vashkevich, 2018^61^	26 (-)	Single-time point	-	Smartphone (with a headset)	-	Distance between vowel envelopes, mutual location of formant frequencies, difference in amplitude of the harmonics.	Norris scale	-	Reduced distance between vowel envelopes in pathology. Harmdiff showed a good separation between control group and ALS. HNR did not show distinction. High accuracy of 88%.
Vashkevich, 2019^60^	15 (-)	Single-time point	-	Smartphone (with a standard headset)	-	Distance between spectral envelopes, formant structure of the speech, formant convergence, breathiness.	None	-	Distance between vowel envelopes, second formant of ‘I’ and second formant convergence of vowels produced good distinction between controls and ALS group. 84.8% accuracy achieved using just second formants of vowel ‘I’.
Vashkevich, 2021^59^	31 (13)	Single-time point	-	Smartphone (with a standard headset)	-	Jitter & shimmer features; F0; spectral envelopes; harmonic-to-noise ratio (HNR); Glottal -to noise excitation ratio (GNE); Mel-frequency cepstral coefficients (MFCC), Phonatory frequency range (PFR), Pitch period entropy (PPE), Pathological vibrato index (PVI) and tremor and harmonics.	None	-	Pathological vibrato index (PVI) and Mel-frequency cepstral coefficients (MFCC) are most valuable. MFCC is valuable for early diagnosis by distinguishing from controls. Pathological vibrato index (PVI) is valuable for identifying later changes and progression of disease. Jitter, shimmer and harmonic-to-noise ratio (HNR) is less useful.
Wang, 2018^46^	12 (-)	Single-time point	-	Microphone	-	Jitter, shimmer and Mel-frequency cepstral coefficients (MFCC)	SIT	-	Combining lip, tongue and acoustic data produces also achieved higher accuracy, better correlation and RMSE than acoustic alone.
Wang, 2016^47^	11 (-)	Single-time point	-	Microphone	-	F0 and Mel-frequency cepstral coefficients (MFCC)	SIT	-	Acoustic data alone produced accuracy above 50%. Lip, tongue, and acoustic data combined improved accuracy to 80.91%. Feasible to detect ALS automatically from short speech samples.
Wang, 2016^48^	9 (-)	Single-time point	-	Microphone	-	F0 features and harmonic-to-noise ratio (HNR)	SIT	-	Feasible with only acoustic data. Adding articulatory data improves model performance.
Weismer, 2001^49^	10 (-)	Single-time point	-	Microphone	-	Formant frequency measure including F2 slopes; intelligibility and speaking rate (SR).	None	-	Total utterance length significantly greater for ALS compared to both PD and controls. Vowel space and F2 slopes taken from either single word or sentence production highly correlated with single word and scaled sentence intelligibility.
Wisler, 2019^50^	66 (-)	24 months	4 sessions with an interval of 4-6 months	Shure Microflex microphone	Shure Microflex microphone	Mel-frequency cepstral coefficients (MFCC).	ALS-FRS(R) SIT	-	Best RMSE and correlations when acoustic data is combined with lip and tongue data using SVR model.
Yunusova, 2016^9^	85 (-)	Single-time point	-	Microphone	-	Speaking rate (SR), articulatory rate (AR) & pause features.	ALS-FRS(R) SIT	33.53 (-)	Articulation rate able to distinguish bulbar disease from respiratory disease. CV phase duration can be used for early detection.

ALS *Amyotrophic lateral sclerosi*s; ALS-FRS(R) *Amyotrophic lateral sclerosis functional rating scale revised,* *ALS-CBS* Amyotrophic lateral sclerosis cognitive behavioural screen, *SIT* Speech Intelligibility Testing, *VC* Vital capacity, *FVC Forced vital capacity,* *SVC* Slow vital capacity, *F0* Fundamental frequency, vF0 Fundamental frequency variation, *Jitter* frequency perturbation, *Shimmer* Amplitude perturbation

### Speech assessment devices (Supplementary Table 1)

24 studies used microphones, 5 used smartphones, 6 used apps, 1 used the Multi-Dimensional Voice Programme (MDVP) and 2 used tape recording devices to record speech. One study provided no information regarding recording of speech samples^24^ and one used two separate databases of speech samples recorded using either a microphone or an app^20^.

The 5 studies using smartphones used the microphone feature of the device alone without the use of a mobile application. Of the 6 studies which did use app-based technology to record speech samples, 3 used the ALS-at-Home app^21,25,26^, one the Beiwe app^27^, one the Help us Answer ALS app^28^ and one the ALS Mobile Analyzer^29^. An additional study used a database of recorded samples to create a prototype of a speech analysis application: the ALS Expert Mobile Application for Android^30^. All studies using apps were published in the last 4 years. 40% of the studies included published from 2019 onwards investigated smartphone related speech assessment.

All studies using microphones and tape recording devices were conducted within a clinic setting^9,16,19,22,23,31–50^, except for one which used an integrated computer system accessed from home via a patient portal or website link. A virtual dialogue agent was then used to instruct participants on speech tasks^51^.

7 studies used sensors in the vocal system in addition to microphones to monitor function of articulatory structures^16,19,40,46–48,50^. Of these, 5 studies (71%) used the Wave Speech Research System, NDI Inc., Waterloo, Canada^19,46–48,50^. This system is an electromagnetic articulograph using sensors placed on the tongue and lip to capture articulatory data. Sensor studies used microphones in addition to simultaneously capture acoustic data^52^.

### Speech tasks (Supplementary Table 2)

Speech sample recording typically involved one or more speech tasks, often preceded by a diagnostic or general neurological examination to determine MND diagnosis and ascertain disease severity. Most of the speech samples were either collected in clinical settings or remotely but using constrained speech elicitation methods. The most common speech tasks were passage reading, used in 12 studies, and sustained vowel phonation, used in 14. Only 4 studies examined free speech using a picture description task^24,34,35,51^ and open ended questions^51^.

### Speech features

Speech feature extraction methods varied and included use of MATLAB in 11 studies, Praat in 10, OpenSMILE in 6, Cspeech in 3, and the Multi-Dimensional Voice Programme (MDVP) in 3. A variety of speech features were assessed, with acoustic features being the most common (30 studies). Frequently extracted features included jitter and shimmer, assessed in 16 studies, and fundamental frequency (F0) evaluated in 23 studies. Other features were assessed less commonly such as pause durations, examined in 13 studies. Fewer studies (16) reported on linguistic features of speech, with the most common feature being speaking rate. There were a variety of ways in which linguistic features were extracted, with a significant portion requiring manual transcription. In the 4 studies that elicited free speech in speech tasks^24,34,35,51^, none assessed the actual content of the language produced.

### Analysis methods

Data analysis methods were equally heterogenous but included the use of R or other statistics packages in 11 studies, MATLAB in 5 studies and CSpeech in 3. Classification models were trained to identify abnormal speech patterns using labelled datasets in 17 (43%) studies. These were then validated on unseen test data. Some of the classification algorithms used included Support Vector Machine (SVM), used in 7 studies, and Linear Discriminant Analysis (LDA), used in 5 studies; and also Decision Tree (DT), Neural Networks (NN), Logistic Regression (LR) and Random Forest (RF)^43,53^. The accuracy, sensitivity, and specificity of the model in differentiating normal speech patterns from those in ALS was then assessed. Alternatively, voice activity detection (VAD) algorithms were employed to detect whether the participant was speaking or not, and evaluated if the system could identify speech periods correctly by accommodating for the speech dysfunction that was present. NEMSI (Neurological and Mental health Screening Instrument), a cloud- based multimodal dialog system, was used to conduct experiments using VAD algorithms in 2 studies^34,35^.

Less frequently, regression analysis was performed to predict a particular continuous outcome, such as intelligible speaking rate^46,47,50^, or predict the total or speech component of ALS-FRS(R) score and the accuracy of the algorithm assessed.

### Comparative measures

15 (38%) studies measured participants’ ALS-FRS(R)^15^, with mean total ALS-FRS(R) scores reported in 11 studies. Bulbar sub-scores were reported in only 4 studies. There were a variety of ways ALS-FRS(R) data was analysed. ALS-FRS(R) was often performed to evaluate disease severity at baseline, monitor progression, as a comparative measure between self-assessed and clinician administered ALS-FRS(R), in addition to being compared against chosen speech assessment device. Other functional assessments such as forced vital capacity (FVC) were conducted by some researchers (details in Table 1).

Cognitive assessments were performed in only 5 studies^9,16,24,27,40^. The ALS Cognitive Behavioural Screen (ALS-CBS) was assessed in one study^27^, however this data was not subsequently reported. The Montreal Cognitive Assessment (MoCA) screening tool was used in 3 studies as a method of excluding participants who did not pass^9,16,40^. Participants with cognitive impairment were excluded from 8 studies^9,16,22,23,32,33,40,42^.

The cognitive-linguistic deficits associated with FTD as opposed to ALS were considered in only two studies. One assessed only pause patterns^9^. The other compared F0, F0 range, mean speech segment duration, total speech duration and pause rate between participants with ALS, ALS-FTD and healthy controls. The Edinburgh Cognitive Assessment Scale (ECAS) and the Mini-Mental State Examination (MMSE) were used to identify participants with ALS-FTD. Scores were subsequently used to identify any associations between acoustic features and cognitive and motor function within ALS and ALS-FTD participant groups^24^.

Other comparative measures included perceptual ratings performed in 15 studies. Perceptual ratings included perceptual scoring by speech language pathologists based on features such as hoarseness, roughness, ‘breathiness’, asthenia, strain, intelligibility, ‘naturalness’ of speech, prosody, voice quality and articulatory precision^32,44^. Where data on perceptual scoring was reported, it was done either at baseline to group participants by disease severity, or as a comparative measure against digital assessment. Speech Intelligibility Testing (SIT), a computerised version of speech intelligibility as a perceptual rating, was performed in 10 studies.

### Risk of bias

**Fig. 2:**
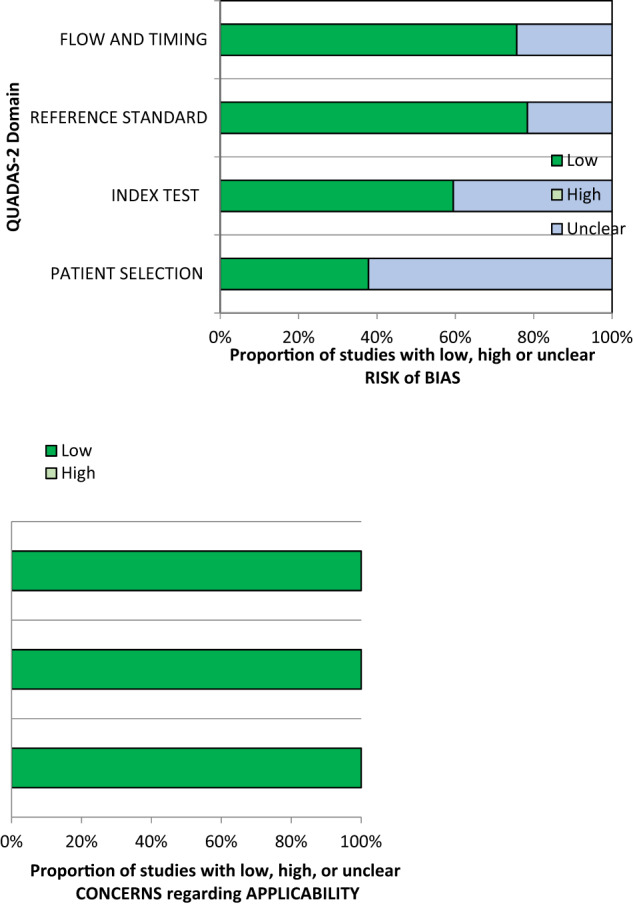
Graphical representation of QUADAS-2 assessment. It depicts the proportion of studies with low (dark green), high (light green) or unclear (blue) risk of bias and applicability concerns.

There was marked heterogeneity across the studies for the index and reference tests used, the methods of analysis and the means of participant recruitment. Similarly, 278 (39%) of responses were defined as “Unclear” due to a lack of information provided by the study in relation to the proposed questions. This affected the ability to draw meaningful conclusions about risk of bias. Domain 2 which assessed patient selection for risk of bias particularly lacked sufficient information. 25 (63%) studies included were found to have an unclear risk of bias regarding patient selection process, higher than any other domain. However, despite missing data, the study populations and proposed devices matched the review question. Thus, applicability concerns were low for all included studies. Graphical representation of the QUADAS-2 assessment is shown in Fig. 2. Full details of risk of bias are shown in Supplementary Table 3.

## Discussion

Our hypotheses included that studies in this field would primarily adopt an exploratory approach, characterised by small sample sizes and limited follow-up duration. Furthermore, we predicted significant variation in the technology used, acoustic features assessed, and speech tasks employed. We anticipated that clinical correlation, if conducted, would be based on established rating scales like the ALS-FRS(R). Consequently, due to this inherent heterogeneity, we expected the clinical utility of the data to be limited.

Synthesis of data extracted from the 40 included papers has largely confirmed the absence of consistency. Results from our comprehensive systematic review indicate that innovative digital speech assessments of speech biomarkers can distinguish between the voices of healthy controls and pwMND and are able to discriminate pwMND from those with other neurological diseases. Nevertheless, short follow-up periods and insufficient data poses challenges in drawing definitive conclusions regarding prognostic implications.

We hypothesised there would be great variation in digital technologies used to assess speech in MND. The use of technology in the 40 included studies was diverse. Although most studies, 24 (60%), used microphones to record speech samples, the tools subsequently employed both to extract and analyse speech features varied (Supplementary Table 1).

The current landscape of speech assessment devices appears to focus on app development providing participants the ability to record speech samples remotely. These can then be analysed either by computer systems or the clinician themselves and are visible on remote databases. A benefit of this approach is that it allows for increased frequency of data collection without imposing the challenges of repeat clinic visits on patients.

However, limited data can be provided by app-based technology regarding kinematic or cognitive elements of speech which are integral to speech function. These are perhaps more able to be assessed within a clinic setting by other device types such as sensors or computer systems. The NEMSI platform with remote dialogue agent did incorporate both acoustic and facial metrics utilising participants’ own computer microphone and camera. High accuracy scores were achieved, suggesting multimodal detection of early changes is possible remotely^51^.

Analysis of collected speech data has typically been conducted using traditional statistical software such as the Systat 6® program^39^, MATLAB^16^, CSpeech^41^ and IBM SPSS Statistics (v.20)^9^. However, ML classification models are increasingly being used to both extract speech data and perform sample analysis. The Montreal Forced Aligner (MFA) and Wav2Vec2 model are examples of algorithms with this capability^38^. Once trained, ML classifiers can correctly label speech data as pathological or non-pathological with significant accuracy^43,53^.

Evidence in this field is emerging and is primarily based upon training classification models to establish the optimum settings for data analysis. No one classifier consistently outperformed others in the included studies, with both SVM and RF exhibiting superior accuracy scores in separate studies^43,53^. Similarly, accuracy scores were lower (84.8%) when discriminating patients with bulbar and non-bulbar ALS, compared to distinguishing controls from bulbar ALS patients where accuracy scores of up to 98.1% were achieved^43^. This would suggest further investigation is needed to achieve ML models that are able to detect early bulbar involvement with a high degree of accuracy. Although data is currently exploratory in nature, the use of more standardised and integrated software which can both extract features and complete data analysis has the potential to streamline data processing of speech samples.

We also predicted a lack of consistency would be reported in terms of assessed speech features. No one feature was assessed in all studies and a diverse range of features were assessed across all included studies. Previous literature has highlighted that the spectral acoustic features of speech, including F0 and formant frequencies, are typical in dysarthria^54^. Changes in jitter and shimmer, in addition to the temporal features of speech, are also known to occur^55^.

Jitter, shimmer and F0 were consistently demonstrated to be higher in MND. These values also changed over time as demonstrated by the significant increase (*p* = 0.001) in jitter, shimmer and F0 seen in participants with ALS at 3 months^10^. Jitter is more uniformly reported to be significantly increased^10,41^. Notably, jitter was included in the most accurate classifier for the ALS Expert Mobile Application for Android app where shimmer was not^30^. It might then be inferred that jitter is a more accurate predictor of dysarthria in MND than shimmer.

Spectral features, including F0 and formant frequencies, were also important in distinguishing pwMND from healthy controls. A smaller F0 range was seen in both ALS and PD, reflective of a more monotonous speech pattern. The F0 range in ALS also appeared narrowed in comparison to healthy controls, but was wider than that of PD^37^. Speech in ALS-FTD also demonstrated a restricted F0 range in comparison with healthy controls and regression analysis showed a strong association between F0 range and the severity of bulbar impairment as measured by a motor examination^24^.

Temporal features were less widely assessed. Previous literature has established that pause durations are increased and SR reduced in pwMND^56,57^. Where assessed, temporal features revealed similar changes^26^. One study was able to achieve good correlations between forced alignment methods and SPA software using temporal related features including pause durations and speech durations. While the study did provide evidence that transformer-based models were viable substitutes for manual speech analysis techniques, the results were achieved using good quality data that might not be reproducible in a clinic or home setting^38^. Overall, no study in this review was able to provide sufficient quality of evidence to support the use of temporal features alone to diagnose or predict bulbar involvement in MND.

Finally, only 5 studies included cognitive assessments as a comparative measure. For the 3 studies which used the MoCA, it was used to exclude participants who did not pass the assessment^9,16,40^. The results of MoCA were only reported in one study, which reported a mean score of 26.44 for pwALS^9^. 30 (81%) studies reported no data relating to the cognitive function of study participants. One study did note the lack of data on cognitive function of trial participants as a limitation^26^.

Language content was not assessed in any study, noted as a limitation of one study^24^. Incorporation of cognitive evaluation into digital speech assessment would give a more comprehensive evaluation of speech dysfunction. Given the influence of cognitive functioning on speech functioning in patients with FTD, research into this area would be useful.

We hypothesised many studies would be exploratory in nature, with a lack of consistency in study procedure and interventions or speech tasks. Synthesis of 40 studies confirmed this lack of consistency. Although use of the Speech Intelligibility Test (SIT)^58^ was common in studies using perceptual analysis as part of the study protocol, there was much diversity in speech tasks used to gather acoustic features for digital analysis. This was largely due to the lack of coherence between extracted speech features. Where the same features were examined, however, similar tasks were used to elicit these. For example, generally passage reading, mainly the Bamboo Passage, was used to elicit temporal measures such as pause duration. Sustained vowel phonation was generally used to elicit F0, jitter and shimmer. Furthermore, ‘a’ was the most analysed vowel. Oral diadochokinesis (DDK) tasks, performed in 9 studies, were conducted by asking participants to repeat syllables as quickly and as accurately as possible^16,19,20,32,34,35,40,42,51^. The specific speech features extracted from this task, however, varied. 5 studies extracted articulatory features such as cycle-to-cycle temporal variation (cTV) and syllable repetition rate (sylRate), speaking and articulation duration^16,19,20,32,40,42,51^; in addition to kinematic features such as tongue movement jitter (movJitter) and alternating tongue movement rate (AMR)^16,19,40^. 2 studies, however, only used recorded speech samples from this task to test voice activity detection algorithms^34,35^.

Furthermore, there were procedural discrepancies across the studies regarding standardisation considerations. Limited data was reported relating to microphone distance with 16 (64%) of studies using microphones giving no information relating to distance from the participant’s mouth at the time of recording. When information was reported, distances ranged from 3 cm to 30 cm^9,33,36,39,41–45,50^, creating challenges both for study comparison and for clinical implementation. Where smartphones were used, 4 studies reported that headsets were used while samples were recorded^30,59–61^ but no other information regarding standardisation was reported.

Location of study also varied. Recording conducted exclusively in a clinic setting provided the advantage of a more controlled recording environment with a greater ability to control confounding factors and standardise experimental design. However, home-based recording of speech samples, using app-based technologies, afforded researchers the ability to follow participants up longitudinally outside of the clinical setting, in addition to enabling participants to self-report their own ALS-FRS(R) questionnaire. However, these studies were preliminary in nature examining raw speech features to assess for abnormality or change over time. Recording of free or spontaneous speech in the wild is advantageous as it allows for participants to record speech unaffected by the clinical environment at a time suited to them. However, it does present the problem of producing samples that cannot be analysed which is a problem to be addressed by future research.

The diagnostic capacity of these technologies and the acoustic features assessed is challenged by the exploratory nature, small sample sizes and limited follow-up periods of many studies within this field. As we initially hypothesised, follow-up periods were consistently short, with 25 (65%) studies being conducted at a single-time-point. The longest duration of follow-up was 60 months occurring in only 2 studies^16,40^. Sample sizes were also small, with a mean number of 41 participants with a diagnosis of MND and a mean number of 15 with bulbar-onset ALS.

With regard to specific speech features, some acoustic features demonstrated decline prior to bulbar function, as assessed by the ALS-FRS(R) or SR. Spectral changes^59^, F0 and DDK^16^, coefficient of variation of phrase^9^ and cycle-to-cycle temporal variation^19^ were each highlighted to be sensitive markers of early bulbar involvement. However, no one feature was consistently highlighted across the studies. Further investigation to confirm these findings for future clinical diagnostic use is needed in addition to robust validation against both perceptual scoring and physical examination by neurologists and speech language pathologists, in combination with specialised tests.

Limited data was available relating to assessment of disease progression due to lack of follow-up. Studies that followed up on participants demonstrated decline in extracted speech features over time^10,26–28,45^. 3 studies reported that rate of decline in assessed acoustic features was faster in bulbar-ALS compared to non-bulbar ALS^16,26,28^. Further longitudinal studies are needed to corroborate these findings.

We initially predicted that if any clinical correlation was done established rating scales like the ALS-FRS(R) would be used. However, limited data relating to clinical correlation was presented. Participants only undertook the ALS-FRS(R) in 15 studies. Of these, correlation of acoustic features with ALS-FRS(R) score was done in only one, where four speech endpoints demonstrated between-patient correlation coefficients of greater than 0.5: average phoneme rate and speaking rate with bulbar domain scores and jitter and shimmer with respiratory domain scores. However, absolute values were more variable with no obvious pattern discernible^23^. One study correlated clinician-based ALS-FRS(R) score with participant rated score to assess for feasibility of at home evaluation^27^, finding that both were equally efficacious. 7 studies used ALS-FRS(R) scores to categorise participants based on disease severity^9,16,22,24,34,35,40^. In 3 studies scores were taken either at baseline or at each session, and either Ridge Regression, SVM or LASSO-LARS were used to predict total ALS-FRS(R) or bulbar sub-scores^28,50,51^. Regression analysis showed promising results of R^2^ values of up to 0.79 for predicting ALS-FRS-R^28^ and high predictive capacity of support vector regression model, *r* = 0.64 for predicting bulbar sub-score^50^. However, all these studies were preliminary and were performed on limited data samples.

The use of perceptual scores also varied. Some studies describe the use of perceptual analysis as part of baseline clinical assessment to assess dysarthria severity while others used it as a speech task. Perceptual scores could also be compared with speech features: Lévêque describes perceptual scoring at baseline to assess disease severity and subsequent individual regression analysis between dysarthria severity, as defined by perceptual score, and speech feature^33^.

Furthermore, no survival-based endpoints were reported in any of the studies. This is an important area of focus for future research if digital assessment of acoustic speech biomarkers is to be used as a potential prognostic marker of bulbar decline.

Integration of appropriately selected acoustic features with digital technology is an area of research with the potential to improve diagnosis and monitoring of pwMND. The additional advantage of remote devices is their ability to offer patients greater access to healthcare with fewer in person clinic visits coupled with increased frequency of data collection. In addition, the use of ML classification models may further streamline data analysis.

For speech assessment technologies to be implemented into clinical practice their validity against existing assessment measures commonly used in MND, such as perceptual rating scales, the ALS-FRS(R), clinical assessments like electromyography (EMG) and nerve conduction studies (NCS), and physical neurological examination would need to be proven.

Exploratory data has found that technology derived scores are highly correlated with clinic ALS-FRS(R) scores^27,50^. Preliminary findings also suggest that digital speech assessment is more sensitive in detecting marginal changes in intensity frequency measures than perceptual analysis, supporting the idea that digital speech biomarkers might aid earlier diagnosis of bulbar involvement before more traditional clinical assessment methods^39^. However, limited data was available relating to this and further investigation is needed.

Acceptability to patients is another important consideration. While the 3 studies reporting on this did conclude that devices were acceptable to participants, improved data collection is required for clinical implementation to be justified^21–23^.

The studies evaluated were largely exploratory. Small sample sizes and lack of participant follow up limited the validity of any conclusions drawn. Missing data meant accurate determination of bias was challenging. A small number of studies meeting inclusion criteria were derived from published conference proceedings. It is possible these were subject to less stringent peer review compared with studies reported in full papers.

Future research should focus on determining the most accurate and sensitive acoustic feature for the assessment of speech in MND. More consistency in the selection of assessment features would help to achieve this. This can then be translated into technology used or device design. Harmonising feature selection and assessment technique would lead to a more substantial and complete evidence base. Additionally, adopting new and innovative techniques into future device design will allow us to integrate complex high-dimensional data like audio into more meaningful information, enabling us to identify new relationships between speech and MND progression. Given that this work has highlighted a shift towards remote and app-based technology, future research should concentrate on increasing the efficiency of remote assessment devices. For example, using edge computing in future at-home devices could provide an option which optimises computing capability, power consumption and speed of data transmission^62^. Furthermore, a considerable number of the studies included in this review reported the use of ML techniques. TinyML is an emerging area of intelligent processing enabling high powered data handling within resource limited devices^63^. By integrating processing within the device itself, in favour of outsourcing to remote servers, clinicians could view patient data in real time creating low power but high performance assessment devices^62^. TinyML has the potential to combine the advantages of ML with those of remote assessment devices. This technique could then be expanded to include assessment of other elements of speech.

Furthermore, while emerging evidence suggests the speech profiles of different neurodegenerative conditions are distinct, there is currently insufficient evidence to substantiate these claims. Further research using larger sample sizes and a greater range of neurodegenerative diseases is needed to clarify any specific speech profile distinctions between MND and other neurodegenerative conditions. Moreover, although speaking rate is recognised as a linguistic aspect of speech, it also serves as an indicator of dysarthria, with dyarthria potentially exerting a more pronounced impact on slowed speaking rate than language or congition in pwMND, depending on how the speech sample is obtained. Interpretation of findings in this systematic review faces challenges in determining the extent of influence from dysarthria, language, and cognitive impairment on speaking rate due to the heterogeneity in study objectives and methodologies across the included studies. Finally, more robust data collection on the acceptability of each device type to patients would also be valuable as this could influence the most appropriate design for use in clinical practice. Patient questionnaires and surveys can be used as effective methods to obtain patient opinion and should therefore be incorporated into future trial design.

Overall, no speech feature alone was consistently able to uniquely identify/diagnose or prognosticate MND. However, the overall speech profile of pwMND was demonstrated to be distinct from that of healthy controls. From the included studies, a complex array of speech features demonstrated changes as MND progresses. Evidence for multi-feature and multimodal approaches to identify and monitor dysarthria in MND is beginning to emerge. Substantial investigation of each approach, with a move towards standardised and harmonised assessment procedures in relation to speech task and a focus on determining the most sensitive speech features will be an important direction for future work.

## Methods

### Search strategy

**Fig. 3:**
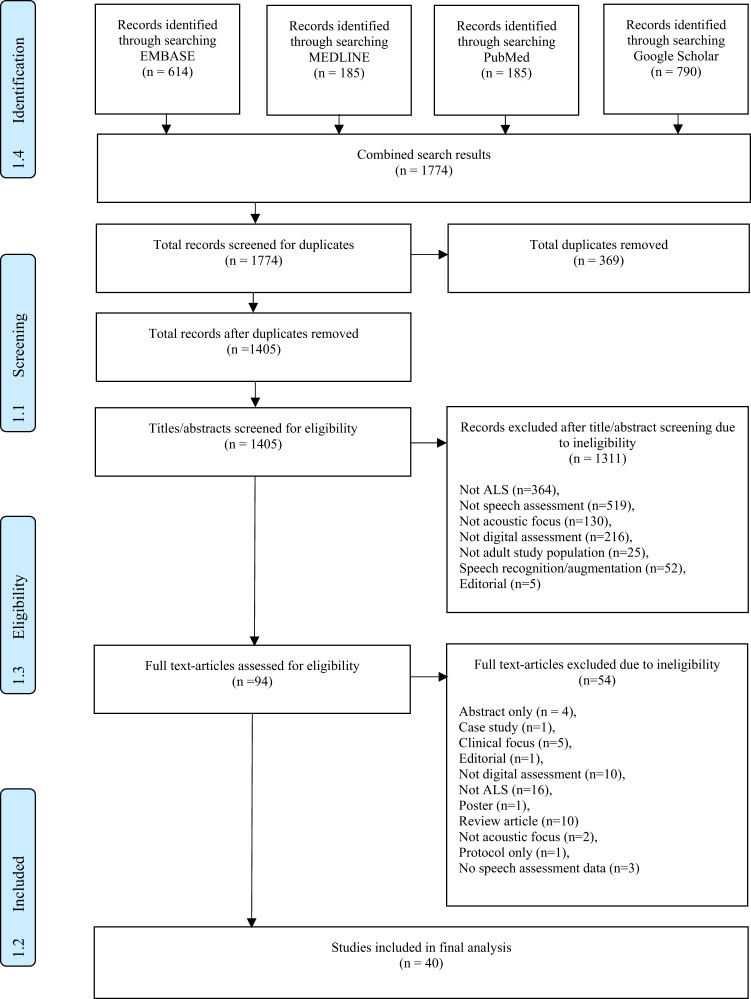
Screening and selection procedure using PRISMA guidelines. From Moher et al.^65^. For more information, visit www.prismastatement.org.

We completed a comprehensive and unbiased systematic literature review on the 4^th^ April 2023. Embase and Medline, were searched using the terms “motor neuron disease” AND “speech”, with the headings exploded to include relevant subheadings including ALS. PubMed was searched using (amyotrophic lateral sclerosis [MeSH Terms]) OR (motor neuron disease [MeSH Terms]) AND (speech[MeSH Terms]). Google Scholar was searched using the terms “amyotrophic lateral sclerosis” OR “motor neuron disease” AND “speech analysis”. No language or date restrictions were applied. Published conference proceedings were also included if they met inclusion criteria. The reference lists of each included result were also assessed for relevant results. The screening process is summarised in Fig. 3: PRISMA Diagram. Because the analysis was based on data from published articles (secondary data), ethical approval and written informed consent from individual participants for this study was not necessary.

A broad search was necessary given the heterogeneity within this research field. The search strategy was developed collaboratively between three authors (M.B, E.B, and S.P). The search terms “technology”, “digital” and “devices” were not included in the final searches as their addition yielded fewer results.

We applied no date restrictions to ensure that no relevant studies were overlooked. However, we anticipated that more recent publications would be more likely to meet our inclusion criteria.

### Study selection

Screening for eligibility was completed independently by two authors (M.B., E.B), with any areas of contention resolved by a third author (S.P.). The inclusion and exclusion criteria are detailed in Table 2.

**Table 2:** Inclusion and exclusion criteria.

Inclusion	Exclusion
Studies involving adults with a diagnosis of MND (any subtype: Amyotrophic Lateral Sclerosis, Progressive Bulbar Atrophy, Primary Lateral Sclerosis, Progressive Bulbar Palsy)	Studies which do not involve patients with a diagnosis of MND (any subtype)
Studies utilising any form of electronic or digital speech assessment to evaluate the acoustic elements of speech, whose output is used in the diagnosis of MND, or to evaluate the progression of MND	Studies involving populations below 18 years of age
Not limited by study design (clinical trials, case-control study, case report, cohort study, case series)	Studies involving animal populations
Not limited by study setting (hospital wards, outpatient clinics, rehabilitation centres, nursing or residential homes or the patient’s own home)	Studies using devices which do not assess speech but another symptom of MND (e.g. spinal symptoms)
	Studies involving electronic speech devices with the purpose of speech augmentation not assessment
	Studies where MND (any subtype) is not the primary cause of speech dysfunction
	Studies involving devices where the acoustic elements of speech are not the main focus of assessment (e.g. those assessing kinematic elements of speech production)
	Review articles

### Quality assessment

Quality assessment was performed using the QUADAS-2 (Quality Assessment of Diagnostic Accuracy Studies) tool^64^. Each study was appraised to have high or low risk of bias in each domain. All studies which fitted inclusion criteria were included in the review regardless of risk of bias.

### Data extraction

Data extraction was performed independently by two authors (M.B and E.B). Information recorded included device used, method of assessment, participant characteristics and any additional assessments conducted. Participant feedback on device suitability was also extracted.

### Supplementary information

Supplementary Information

Project Data
