# Supplementary material for: A systematic review and narrative analysis of digital speech biomarkers in Motor Neuron Disease
Source: NPJ Digit Med. 2023 Dec 7;6:228. doi: 10.1038/s41746-023-00959-9 (PMC10703929; doi:10.1038/s41746-023-00959-9)
Supplement: Supplementary file 1 — Supplementary Information [file 41746_2023_959_MOESM1_ESM.pdf]

Supplementary Information

Supplementary Table 1: Summary of digital speech assessments technologies

First Author and Publication Year	Devices described in methods	Device Brands	Speech sample acquisition method	Speech feature extraction method	Data analysis method	Was machine learning used?	Was machine learning used for classification, regression, or both?
Agurto, 2019 [28]	Smartphone App	Help us Answer ALS	App	Praat toolkit used for all except ASR. Automatic IBM Speech to text tools.	NIST Scoring Toolkit for ASR. R2. Machine learning methods.	Yes	Both
Berry, 2019 [27]	Smartphone App	Beiwe	App	Pre-processed using Audacity 2.2.2. Speech Pause Analysis (SPA) software, a MATLAB speech pause segmentation procedure.	R version 3.5	No	-
Buder,1996 [31]	Audiocassette & microcomputer-based analysis program	CSpeech	Audiocassette	C-Speech	IBM-compatible microcomputer-based analysis program (CSpeech) to analyse speech signals.	No	-
Cebola, 2023 [53]	Smartphone	-	Smartphone	Pre-processing-WebRTC's voice activity detection (VAD) using Gaussian Mixture Models (GMM). Feature extraction: Window-based features extracted using Time Series Feature Extraction Library (TSFEL); other features extracted using Praat (Parselmouth library used to	The ML classifiers selected for this task were Support Vector Machines (SVM), Logistic Re-gression (LR), Naive Bayes (NB), Decision Tree (DT) and Random Forest (RF)	Yes	Classification

				compute Praat features in Python).			
Chiaramonte, 2019 [10]	Integrated computer software	Multi-Dimensional Voice Programme (MDVP)	Multi-Dimensional Voice Programme (MDVP)	Multi-Dimensional Voice Programme (MDVP)	Multi-Dimensional Voice Programme (MDVP)	No	-
Garcia-Gancedo, 2019 [22]	Microphone	High fidelity speech capture system	Microphone	offline using a MATLAB	MATLAB	No	-
Kelly, 2020 [23]	Microphone and computer	High fidelity speech capture system	Microphone	offline using a MATLAB	MATLAB	No	-
Laganaro, 2021 [32]	Microphone and computer	MonPaGe Protocol	Microphone	Praat	Praat and R	No	-
Lévêque, 2022 [33]	Microphone	Focusrite Scarlett (2i4) external audiocard and a professional quality Shure SM35-XLR earset microphone	Earset Microphone	Praat	R (Version 4.0.2)	No	-
Likhachov, 2021 [30]	Smartphone App	ALS Expert Mobile Application for Android.	Smartphone	linear discriminant analysis (LDA) classifier model	linear discriminant analysis (LDA) classifier model	Yes	Classification
Liscombe, 2021 [35]	Microphone and computer dialogue system	NEMSI	Microphone	NEMSI (NEurological and Mental health Screening Instrument)/ Voice activity detection using	NEMSI (NEurological and Mental health Screening Instrument)/	Yes	Classification

				the CMU Sphinx open source speech recognition toolkit	Voice activity detection using the CMU Sphinx open source speech recognition toolkit		
Liscombe, 2023 [34]	Microphone and computer dialogue system	NEMSI	Microphone	NEMSI (NEurological and Mental health Screening Instrument)/ Voice activity detection using the CMU Sphinx open source speech recognition toolkit	NEMSI (NEurological and Mental health Screening Instrument)/ Voice activity detection using the CMU Sphinx open source speech recognition toolkit. NIST Detection Cost Function (DCF)	Yes	Classification
Maffei, 2023 [36]	Microphone	Audio-Technica AT831R	Lapel Microphone	Sustained vowel- Praat; Passage reading- The Loudness Normalization function in Audacity (Audacity Team, 2021)	pROC and ROCR packages in R.	No	-
Mori, 2004 [37]	Microphone	Dynamic microphone or electret condenser microphone MI-1233	Microphone	Window length method (F0 range), ARX speech analysis method (vowel formant frequencies), error correction performed manually and by visual inspection	Window length method (F0 range), ARX speech analysis method (vowel formant frequencies), error correction performed manually and by visual inspection	No	-

Naeini, 2022 [38]	Microphone and computer programme	Montreal Forced Aligner (MFA) and pre-trained transformer based Wav2Vec2 model.	Microphone	Pre-processing- semi-automatic Speech and Pause Analysis (SPA) software. Two forced aligner methods: Montreal Forced Aligner (MFA) and transformer-based Wav2Vec2 model.	Two forced aligner methods: Montreal Forced Aligner (MFA) and transformer-based Wav2Vec2 model	Yes	Classification
Neumann, 2021 [51]	Computer camera and microphone	"Nina"- remote dialogue agent	Microphone	Praat	Traditional statistics, R2, Binary classifcation, LASSO-LARS-regression	Yes	Both
Nevler, 2020 [24]	Computer systems	Speech Activity Detector (SAD), developed at the University of Pennsylvania Linguistic Data Consortium.	-	Automatically segmented using Speech Activity Detector (SAD) and visually reviewed. Pitch tracking performed using Praat.	Traditional statistics including Pearson correlation, univariate linear regression models, univariate multiple regression analysis.	No	-
Norel, 2018 [29]	Smartphone App	ALS Mobile Analyzer	App	OpenSMILE toolkit.	Classifiers including linear upport vector machines (SVM), decision tree, and regression analysis	Yes	Classification
Peplinski, 2019 [25]	Smartphone App	ALS at Home	App	Pre-processing- voice activity detector (VAD); Praat	SMOTE (Synthetic Minority Over-Sampling Technique), logistic regression classifiers	Yes	Classification

Robert, 1999 [39]	Digital tape recorder and computer voice analysis system	EVA®	Digital tape recorder	EVA objective analysis system	Systat 6® pro- gram	No	-
Rong, 2015 [16]	Microphone and sensor	Countryman E6 microphone. Multidimensional Voice Programme (MDVP)	Microphone	Phonatory subsytem-Multidimensional Voice Programme (MDVP, Model 5105) software; Respiratory subystem-Phonatory Aerodynamic System (PAS); Articulatory subsystem-SMASH, a custom MATLAB program; Resonatory subsystem-nasometer and PAS	MATLAB program (MATLAB R2013b) Speech Pause Analysis (SPA)	No	-
Rong, 2016 [40]	Microphone and sensor	Countryman E6	Microphone	Phonatory subsytem-Multidimensional Voice Profile (MDVP, Model 5105) software; Respiratory subystem-Phonatory Aerodynamic System (PAS); Articulatory subsystem-SMASH, a custom MATLAB program; Resonatory subsystem-nasometer and PAS	MATLAB program (MATLAB R2013b) Speech Pause Analysis (SPA)	No	-
Rong, 2020 [19]	Microphone and sensor	Wave system, NDI Inc	Microphone	MATLAB	MATLAB and R statistical analysis program	No	-
Rowe, 2022 [20]	Microphone and Smartphone App	Professional quality microp	Microphone or App (depending on	Segmented manually and formant tracking validated	R (R Core Team 2014)	No	-

		hones (e.g., AKG C410, Shure SM81 Condenser, Olympus VN-702PC digital recorder) or the Beiwe application	database used)	through visual inspection. Feature extraction using custom Praat script and the features were subsequently calculated using custom MATLAB (MathWorks 2019) and R scripts.			
Rutkove, 2020 [21]	Smartphone App	ALS at home	App	-	-	No	-
Silbergleit, 1997 [41]	Microphone and computer system	Cspeech CompuAdd computer, model 320/325 IBM ACPA (audio capture and playback adapter) A/D D/A card	Headband microphone	Cspeech waveform analysis	Cspeech waveform analysis	No	-
Stegmann, 2020 [26]	Smartphone App	ALS at home	App	Statistical model-based voice activity detector	R: packages lme4 and nlme.	No	-
Tanchip, 2022 [42]	Microphone	Marantz PMD660 compact flash recorder with an accompanying Countryman E6 omnidir	Microphone	Manual segmentation. Automated segmentation using Five algorithms used for automated segmentation of DDK data were used: Novotny, Neurospeech (VAD in Praat), DTA, Rong, Energy (MATLAB).	Manual segmentation. Automated segmentation using Five algorithms used for automated segmentation of DDK data were used: Novotny, Neurospeech	No	-

		ectional microphone or an Olympus WS-853 recorder with an accompanying ME52W unidirectional microphone.			(VAD in Praat), DTA, Rong, Energy (MATLAB). Statistical analyses to assess interrater reliability, receall and precision, and concurrent validity of the automated tools was done on R (RStudio Team, 2020) and open-source R script (with the nlme package).		
Tena, 2022 [43]	Microphone and computer software	USB EMITA Streaming GXT 252 microphone and Audacity (open-source application).	Microphone	Recorded using Audacity. Extracted using MATLAB.	Classification algorithms: Support Vector Machine (SVM), Neural Networks (NN), Linear Discriminant Analysis (LDA), Logistic Regression (LR) and Random Forest (RF)	Yes	Classification
Tomik, 2015 [44]	Microphone and computer software	IRIS Otolaryn-gologia 2004 software	Microphone (computer microphone)	IRIS Otolaryn-gologia 2004 software,	STATISTICA v. 8.0 PL	No	-
Tomik, 1999 [45]	Microphone and computer software	Bruel and Kjaer microphone and transducer	Microphone	Spectograms prepared using spectographs. Micro-computer. speech analysis program	Spectograms prepared using spectographs. Micro-computer. speech analysis	No	-

		Computer PC 48 program		(computer PC 486)	program (computer PC 486). Traditional statistical analysis.		
Vashkevich, 2018 [61]	Smartphone	-	Smartphone (with a standard headset)	Linear predictive analysis (traditional algorithms), harmonic analysis	linear discriminant analysis (LDA) classifier model using the FisherCcriterion	Yes	Classification
Vashkevich, 2019 [60]	Smartphone	-	Smartphone (with a standard headset)	Dynamic time warping (DTW) algorithm-vowel extraction; LPC analysis-formant features	Linear discriminant analysis (LDA) classifier with Fisher Criterion	Yes	Classification
Vashkevich, 2021 [59]	Smartphone	-	Smartphone (with a standard headset)	Feature selection algorithms: (1) maximization of quality of variation (QoV), (2) Relief (3) least absolute shrinkage and selection operator (LASSO), (4) RelieFF	linear discriminant analysis (LDA) with Fisher criterion	Yes	Classification
Wang, 2018 [46]	Sensors	Wave Speech Research System, Northern Digital Inc., Waterloo, Canada	Microphone	Feature extraction using OpenSMILE; Feature selection using Gradient Boosting	Support Vector Machine Regression	Yes	Regression
Wang, 2016 [47]	Sensors	NDI Wave and Carstens EMA AG500	Microphone	Feature Extraction: OpenSMILE; Feature selection: Randomized Logistic Regression (RLR)	Support Vector Machine (SVM); Deep Neural Network (DNN)	Yes	Classification

Wang, 2016 [48]	Sensors	Wave system, NDI Inc., Waterloo, Canada	Microphone	Feature extraction using OpenSMILE; Feature selection using Decision Tree and Gradient Boosting	Support Vector Machine (SVM)	Yes	Both
Weismer, 2001 [49]	Tape recorder and computer programme	CSpeech	Microphone	CSpeech	CSpeech	No	-
Wisler, 2019 [50]	Sensors	NDI Wave System (Northern Digital Inc., Waterloo, Canada)	Shure Microflex microphone	SMASH, a Matlab based software	Ridge Regression and Support Vector Machine (SVM)	Yes	Regression
Yunusova, 2016 [9]	Microphone	-	Microphone	Preprocessing-Adobe Audition1 (version 2.0); Speech Pause Analysis (SPA) software, a semi-automated MATLAB speech pause segmentation procedure	IBM SPSS Statistics (v.20)	No	-

Supplementary Table 2: Speech tasks and acoustic features

First Author and Publication Year	Domains assessed			Speech tasks	
	Acoustic	Para-linguistic	Linguistic	Speech tasks carried out	Acoustic feature elicited from each task
Agurto, 2019 [28]	Yes	Yes	Yes	Passage reading, describing a picture, counting until runs out of breath	Unclear which task- Compute the speech rate and articulation rate, voiced/unvoiced ratio, and voiced/unvoiced percentages in the speech.<br>Automatic speech recognition accuracy taken from reading task.

Berry, 2019 [27]	No	Yes	No	Active: passage reading (Bamboo passage) and production of cough. Answering ALSFRS-R. Passive: communication logs.	Passage reading- pause measures including mean pause duration. Exploratory analysis of the cough spectrograms obtained is planned but has not yet been performed.
Buder,1996 [31]	Yes	Yes	No	Sentence production- 'The potato stew is in the pot'.	Sentence production for all speech features
Cebola, 2023 [53]	Yes	Yes	No	Sentence repetition, cough production, and sustained vowels task /u/, /a/ and /i/, thrice recorded at a comfortable pitch and loudness for as long as possible.	Sentence phonatory task- silence features. Sustained vowel and sentence task- formant features. All tasks- window based features.
Chiaramonte, 2019 [10]	Yes	Yes	No	Spontaneous speech evaluated during a medical examination. Repetition of long sentences and maintained vowel throughout expiration after forced inspiration, phoneme repetition.	Sentence repetition and sustained vowel task- pneumophonic coordination, Maximum Phonation Time (MPT) and ability to pronounce /i/u/e/ consecutively. Phoneme repetition- the pronunciation of explosive consonants /p/ t/ k/ b/ d/ g/, velar consonants /k/ g/ and lingual consonants /l/ r/. On physical examination- oral dysdiadochokinesia and fasciculations by evaluating articular structures (lips, tongue, jaw), velopharyngeal structures (glosso-palatal and velar-pharyngeal sphincter), phonation structure (laryngeal morphology and motility).
Garcia-Gancedo, 2019 [22]	Yes	Yes	Yes	4 speech tests: repetition of "Ah" 7x; sustained "Ah" 10seconds; pronounce "Doily" 3x; passage reading (bamboo passage)	Test 1: Long "Ah"- F0, jitter and shimmer. Test 2: Short (repeat) "Ah"- F0, jitter, shimmer. Test 3: "Doily" - Average 'oi' phoneme rate, Maximum 'oi' phoneme time. Test 4: Bamboo Passage- Pause Time and Speaking Rate.
Kelly, 2020 [23]	Yes	Yes	Yes	4 speech tests: repetition of "Ah" 7x; sustained "Ah" 10seconds; pronounce "Doily" 3x; passage reading (bamboo passage)	Test 1: Long "Ah"- F0, jitter and shimmer. Test 2: Short (repeat) "Ah"- F0, jitter, shimmer. Test 3: "Doily" - Average 'oi' phoneme rate, Maximum 'oi' phoneme time. Test 4: Bamboo Passage- Pause Time and Speaking Rate.
Laganaro, 2021 [32]	Yes	No	Yes	A Composite Perceptual Score- computed based on	A Composite Perceptual Score- computed based on a

				a perceptual rating of the participant's speech on five dimensions: voice quality, segmental realization, prosody, intelligibility, and naturalness of speech.<br>intelligibility test was administered in the form of an interactive task between the experimenter and the participant in a face-to-face setting.<br>Articulation task: assessed on the production of a set of 50 pseudo-words (covering most French consonants and vowels as well as consonant clusters).<br>Sustained vowel phonation of /a/ for as long as possible after taking a maximal inhalation, at a comfortable pitch and at their habitual loudness.<br>Sustained production for 2–3 seconds of the vowel/ a/at a comfortable height and loudness.<br>Sentence production- reading of a 7-syllable sentence composed of only voiced sounds ("Mélanie vend du lilas" – [melanivɑ̃dylila], 'Melanie sells lilac').<br>Production of a 4 syllable sentence- sentence 'Laurie l'a lu' ([loʁilaly], 'Laurie read it').<br>short sentence reading task- - "Mélanie vend du lilas".<br>Oral diadochokinesis (DDK) task- 7 items varying in phonological complexity. Participants were instructed to produce these sequences in a continuous manner for at least five second as fast and as accurately as possible.	perceptual rating of the participant's speech on five dimensions: voice quality, segmental realization, prosody, intelligibility and naturalness of speech.<br>Intelligibility task - intelligibility<br>Articulation task- articulatory precision.<br>Sustained vowel task- MPT.<br>Sustained vowel task (2-3s) and sentence production- voice-related measures (jitter, shimmer, cepstral measures.<br>4 syllable sentence- prosodic contrast (calculated as difference in f0 modulation).<br>Short sentence reading task- speech rate.<br>DDK task – DDK rate and a sequential motion rate (SMRCV).
Lévêque, 2022 [33]	Yes	Yes	Yes	Perceptual Score (The severity of dysarthria assessed using Batterie d'Evaluation Clinique de la Dysarthrie, BECD, using intelligibility,	Perceptual Score - intelligibility, naturalness of speech, prosody, voice quality and articulatory precision

				naturalness of speech, prosody, voice quality and articulatory precision).<br>Syllable repetition- produced 3 sequences of syllables either vowel-glide or glide-vowel.<br>Sentence repetition- repeated each sentence 4 times (total 12 sequences per participant).	TSC_MFCC contour was taken from the steady part of the first segment (i.e., the first<br>valley) to the steady part of the last segment (i.e., the last valley).<br>Mean and VARCO extracted from all sentences.<br>eventDUR intervals from the acoustic transition between one segment to the next in the /ajajaj/, /ujujuj/, and /wiwiwi/ sequences.
Likhachov, 2021 [30]	Yes	No	No	Sustained vowel task- pronunciation of /a/ for as long as possible.	Sustained vowel task- all features.
Liscombe, 2021 [35]	No	Yes	No	An open-ended question about difficulty in speaking, salivating, swallowing.<br>Sustained vowel phonation of /A/.<br>Oral Diadochokinesis Alternating Motion Rate (DDK AMR) or repetition of the syllables /pAtAkA/ (DDK).<br>Speech Intelligibility Test sentences (SIT).<br>Passage reading about (bamboo passage).<br>Spontaneous speech while describing a picture.	All features from all tasks.
Liscombe, 2023 [34]	No	Yes	No	Sustained vowel phonation, counting diadochokinetic syllables, read short sentences and a longer paragraph, spontaneous speech, and description of a picture.	All features from all tasks.
Maffei, 2023 [36]	Yes	No	No	Sustained vowel task- /a/ for as long as possible in one breath.<br>Reading Bamboo passage aloud.<br>Perceptual assessment- Three of the authors who are speech-language pathologists independently rated the phonatory quality using a adapted from CAPE-V.	Sustained /a/- local jitter, local shimmer, HNR, Cepstral/spectral measures<br>Continuous speech sample/passage- Cepstral/spectral measures<br>Phonatory quality by perceptual assessment of all tasks.
Mori, 2004 [37]	Yes	No	No	Passage reading. Each subjects read an Aesop story “The North Wind and The Sun” (8 sentences) or “Sakura” passage (8 sen- tences),	F0 range and F0 minimimum extracted from whole recorded sentences.<br>Vowel formant frequencies extracted automatically from whole utterances.

				depending on recording date.	
Naeini, 2022 [38]	No	Yes	Yes	Passage reading- Bamboo passage.	Passage reading- all tasks.
Neumann, 2021 [51]	Yes	Yes	Yes	Sustained vowel phonation.<br>Read speech- the dialog contains six speech intelligibility test (SIT) sentences of increasing length (5 to 15 words), and one passage reading task (Bamboo Passage; 99 words).<br>Oral Diadochokinesis (DDK) task-measure of diadochokinetic rate (rapidly repeating the syllables /pAtAkA/).	Sustained vowel- Mean F0 (Hz), jitter (%), shimmer (%), HNR (dB), CPP (dB); SIT and Passage reading- Speaking and articulation duration (sec), speaking and articulation rate (words/min), percentage pause time (PPT).<br>DDK- Speaking and articulation duration (sec), Syllable rate (syllables/sec), number of produced syllables, cycle-to-cycle temporal variation (cTV) (sec).
Nevler, 2020 [24]	Yes	No	Yes	Free speech task- describing the Cookie Theft picture.	All features from picture description task.
Norel, 2018 [29]	Yes	No	No	3 sentences or 1 paragraph in English at their own pace at a preferred location. (Not participants were native English speakers, and not all read all the suggested sentences.)	No data
Peplinski, 2019 [25]	Yes	No	No	Sustained vowel phonations of /a/.<br>Perceptual assessment- Seven phonations from each ALS patient were assessed for tremor by a speech language pathologist (SLP).	All features from sustained vowel phonation.
Robert, 1999 [39]	Yes	No	No	Sustained vowel phonations /a/.<br>Perceptual voice assessment was also performed by speech language pathologist (SLP).	All features from sustained vowel phonation.
Rong, 2015 [16]	Yes	Yes	Yes	Syllable repetition 7x maintaining constant pitch & loudness.<br>Normal and high pitch phonation of /a/.<br>Oral Diadochokinesis (DDK) rate test.<br>Sentence reading.<br>Sentence Intelligibility Testing.	Respiratory subsytem: /pa/, /pi/<br>Bamboo passage- Maximum subglottal pressure, speech duration, pausing pattern (e.g., number of pauses, pause duration, pausing frequency).<br>Phonatory: “Normal” and “high pitch” phonation of /a/- Phonation duration, maximum F0, jitter,

					shimmer, NHR, SPL, laryngeal airway resistance.<br>Articulatory: “Buy Bobby a puppy”, “Say /aCa/ again”. Repeat /ba/ as clear and as fast as possible on one breath- Maximum/minimum velocities of lips and jaw Number, duration, and rate of syllable repetitions.<br>Resonatory: “Mama made a lemon jam” “Buy Bobby a puppy”, /pa/, /pi/, /ma/, /mi/, “hamper”- Nasalance, Intraoral air pressure and nasal airflow in syllables, time lag between /m/ and /p/ in “hamper”
Rong, 2016 [40]	Yes	Yes	Yes	Syllable repetition 7x maintaining constant pitch & loudness.<br>Normal and high pitch phonation of /a/.<br>Oral Diadochokinesis (DDK) rate test.<br>Sentence reading.<br>Sentence Intelligibility Testing.	Respiratory subsytem: /pa/, /pi/<br>Bamboo passage- Maximum subglottal pressureSpeech duration, pausing pattern (e.g., number of pauses, pause duration, pausing frequency).<br>Phonatory: “Normal” and “high pitch” phonation of /a/- Phonation duration, maximum F0, jitter, shimmer, NHR, SPL, laryngeal airway resistance.<br>Articulatory: “Buy Bobby a puppy”, “Say /aCa/ again”. Repeat /ba/ as clear and as fast as possible on one breath- Maximum/minimum velocities of lips and jaw Number, duration, and rate of syllable repetitions.<br>Resonatory: “Mama made a lemon jam” “Buy Bobby a puppy”, /pa/, /pi/, /ma/, /mi/, “hamper”- Nasalance, Intraoral air pressure and nasal airflow in syllables, time lag between /m/ and /p/ in “hamper”
Rong, 2020 [19]	No	Yes	Yes	At baseline speech intelligibility and speaking rate taken from -sentence repetition, a word identification test consisting of 54 words and sentences taken from a passage.<br>Procedure- oral diadochokinesis task- syllable repetition /ta/ at	At baseline XRMB database:<br>Speaking rate taken from “To feed the cat one must shoo the dog”.<br>Word intelligibility- word identification test consisting of 54 words (Ten listeners performed this test, and the average percentage of correctly identified words for each speaker across the

				maximum rate in one breath.	10 listeners was derived as WordIntell). Scaled sentence intelligibility- listeners rated the intelligibility of two sentences from the Hunter Passage (i.e., “Tom Brooks was such a man; and Once he thought he saw a bird, but it was a large leaf that had failed to drop to the ground during the winter”) and an additional sentence (i.e., “To feed the cat one must shoo the dog”. At baseline SSD database: Intelligibility and speaking rate obtained from the SIT (participants read 11 randomly generated sentences of five to 15 words. Their speech was transcribed by two naïve listeners). Procedure: Oral dadochokinesis (DDK) task- cycle-to-cycle temporal variation (cTV) and syllable repetition rate (sylRate). Kinematic measures tongue movement jitter (movJitter) and alternating tongue movement rate (AMR) taken from sensor data during DDK task.
Rowe, 2022 [20]	Yes	Yes	Yes	Perceptual assessment- Two licensed speech-language pathologists with clinical expertise in speech motor disorders rated each speaker’s level of articulatory severity on a categorical scale of "Normal," "Mild," "Moderate," "Severe," or "Profound". Categorical ratings were used to stratify the speakers and those with “severe” or “profound” impairment were excluded. The clinicians were also asked to rate each speaker on a 100-point visual analogue scale with endpoints labelled "No Impairment" (0) on the left and "Profound Impairment" (100) on the right.	All taken from SMR task. Coordination (GapSyllProp)- relative duration of the silence between two articulatory gestures during each syllable transition. Consistency (RepVarVOT)- Across-repetition variability in voice onset time. Speed (F2Slope)- Second formant slope in the consonant transition of /k. Precision (ConVarF2Slope)- Across-consonant variability in second formant slope in the consonant transitions of /p/, /t/, and /k/. Rate (RepRate)- number of syllables produced per second.

				Sequential motion rate (SMR) task, in which participants were instructed to repeat /pataka/ as quickly and accurately as possible on one breath.	
Rutkove, 2020 [21]	-	-	-	-	-
Silbergleit, 1997 [41]	Yes	No	No	Sustained vowel /a & i/ phonation at comfort pitch, low pitch, and high pitch for 3s. Subjects followed the same protocol of producing three repetitions of both sounds, at three pitches.	Taken from both vowels at all pitches- jitter, shimmer, maximum phonation frequency range (MPFR) and signal-to-noise ratio (SNR).
Stegmann, 2020 [26]	No	No	Yes	Reading of 5 sentences.	Sentence reading- articulatory precision (AP) and speaking rate (SR)
Tanchip, 2022 [42]	No	Yes	Yes	SIT (participants read 11 randomly generated sentences containing 15 words- naïve adult listener transcribed.<br>DDK- repeated the syllables /ba/, /pa/, or /ta/ as fast as possible on one breath. Each task was repeated up to 2 times; only the second repetition was used for analysis. Some participants performed only one of the three tasks; others repeated both /ba/ and /ta/, or both /pa/ and /ta/. Thirty-eight participants performed only /ba/, 45 only /pa/, 40 only /ta/, 21 /ba/ and /ta/, and 12 /pa/ and /ta/.	SIT- Speaking rate was measured as the average number of words produced per minute (words per minute [WPM]), and intelligibility was measured as the proportion of correctly transcribed words to the total amount of words across sentences.<br>DDK task- cTV, DDK rate, no of syllables.
Tena, 2022 [43]	Yes	Yes	No	Sustained vowel- sample of each Spanish vowel for 3–4s.	All features from sustained vowel.
Tomik, 2015 [44]	Yes	Yes	No	Perceptual assessment performed independently by 2 assessors voice graded using GRBAS (all features except for hoarseness scaled normal (1) or abnormal (2).<br>Videolaryngostroboscopy (VLS). Acoustic analysis using sustained vowel phonation "a".	Perceptual assessment- grade of hoarseness, roughness, breathiness, asthenia, and strain.<br>VLS- voice range and maximum phonation time (MPT), symmetry and regularity of vocal folds vibration; amplitude of vibrations; mucosal wave, reflecting the shift of the mucosa in relation to the vocal muscle movement during phonation; glottic

					closure, and immobility of vocal folds. Acoustic analysis- mean fundamental frequency of voice, expressed in Hz (F0); relative cycle-to- cycle variation of F0, expressed in % (jitter); relative cycle-to-cycle perturbation of harmonic amplitude with the frequency equal to F0, expressed in % (shimmer), and relation of the nonharmonic (noise) to harmonic reflecting the amount of noise in the voice signal, expressed in % (noise-to-harmonic ratio (NHR)).
Tomik, 1999 [45]	No	Yes	Yes	Repetition of a test sound 3x. Repetition of sentence 3x.	These consonants and vowels were analysed alone and in a standard Polish sentence: R, L, D, T, M, W, P, B, G, K, H, Q, O, U, I. The sentence: *w calym kraju dzis jest ladna pogoda* (*in whole country is fine weather)* was used.
Vashkevich, 2018 [61]	Yes	Yes	No	Running speech test counting 1-10. Vowels /æ/ and /i analysed.	Running speech test- distance between vowel envelopes, mutual location of formant frequencies, difference in amplitude of the harmonics. Fragments of speech signal containing the vowels /æ/ and /i/, from the words “odin”, “dvæ”, “tri” (one, two, three).
Vashkevich, 2019 [60]	Yes	No	No	Running speech test- sentence counting 1 to 3 in Russian “ædin, dvæ, tri”- /æ/ and /i/ vowel analysis	Running speech test- distance between vowel envelopes, mutual location of formant frequencies, difference in amplitude of the harmonics. Fragments of speech signal containing the vowels /æ/ and /i/, from the words “odin”, “dvæ”, “tri” (one, two, three).
Vashkevich, 2021 [59]	Yes	No	Np	Sustained vowel task- /a & i/ phonation at comfortable pitch & loudness for as long as possible.	All features extracted from sustained vowel phonation task.
Wang, 2018 [46]	Yes	No	No	Repetition of 20 common phrases sequentially. Speech intelligibility testing by a certified Speech-Language Pathologist.	Phrase repetition- Intelligible Speaking Rate Prediction and acoustic features including Jitter, shimmer & mel frequency cepstral coefficients (MFCC).

					Speech intelligibility and speaking rate were measured by a certified Speech-Language Pathologist using the software Sentence Intelligibility Test (SIT)
Wang, 2016 [47]	Yes	No	No	Sentence repetition (e.g., how are you doing?).<br>8 isolated vowels /bVb/ form (i.e., /bab/, /bib/, /beb/, /b@b/, /bˆb/, /bcb/, /bob/, /bub/) .<br>Speech intelligibility testing.	Sentence and vowel repetition- Jitter, shimmer, F0, mel frequency cepstral coefficients (MFCC).<br>The speech intelligibility and speaking rate of patients were evaluated by a certified speech-language pathologist using the Sentence Intelligibility Test (SIT) software.
Wang, 2016 [48]	Yes	No	No	Phrase repetition.<br>SIT.	Phrase repetition- Speech intelligibility prediction using features including jitter, shimmer, F0 features and harmonic to noise ratio (HNR).<br>The speech intelligibility and speaking rate of these patients were evaluated by a certified speech-language pathologist us- ing the Sentence Intelligibility Test (SIT) software
Weismer, 2001 [49]	Yes	No	Yes	Sentence repetition (6 repeated 6 times each).<br>Intelligibility- perceptual assessment using direct magnitude estimation (DME).	Temporal measures- Selected segment durations and total utterance.<br>Spectral measures consisted of formant frequency measures for the corner vowels, and F2 slopes derived from the /aɪ/ in ‘buy’ for example.<br>Intelligibility- perceptual assessment using direct magnitude estimation (DME).
Wisler, 2019 [50]	Yes	No	No	Sentence production (20 sentences in a fixed order).<br>Sentence Intelligibility Test (SIT).	Speech intelligibility and speaking rate were assessed by a speech-language pathologist using the Sentence Intelligibility Test (SIT) software.<br>Sentence production- mel frequency cepstral coefficients (MFCC).
Yunusova, 2016 [9]	No	Yes	Yes	Passage reading- 60 word paragraph (Bamboo passage)	All features extracted from passage reading.

Supplementary Table 3: Tabular Representation of QUADAS-2

STUDY	RISK OF BIAS				APPLICABILITY CONCERNS		
	PATIENT SELECTION	INDEX TEST	REFERENCE STANDARD	FLOW AND TIMING	PATIENT SELECTION	INDEX TEST	REFERENCE STANDARD
Agurto, 2019 [28]	?	☺	?	?	☺	☺	☺
Berry, 2019 [27]	?	?	☺	☺	☺	☺	☺
Buder,1996 [31]	?	?	?	?	☺	☺	☺
Cebola, 2023 [53]	?	?	☺	?	☺	☺	☺
Chiaramonte, 2019 [10]	☺	?	☺	☺	☺	☺	☺
Garcia-Gancedo, 2019 [22]	☺	☺	☺	☺	☺	☺	☺
Kelly, 2020 [23]	☺	☺	☺	☺	☺	☺	☺
Laganaro, 2021 [32]	?	☺	☺	☺	☺	☺	☺
Lévêque, 2022 [33]	☺	?	☺	☺	☺	☺	☺
Likhachov, 2021 [30]	?	☺	?	?	☺	☺	☺
Liscombe, 2021 [35]	?	☺	☺	☺	☺	☺	☺
Liscombe, 2023 [34]	?	?	☺	?	☺	☺	☺
Maffei, 2023 [36]	☺	?	☺	☺	☺	☺	☺
Mori, 2004 [37]	☺	☺	?	☺	☺	☺	☺
Naeini, 2022 [38]	?	?	☺	?	☺	☺	☺
Neumann, 2021 [51]	☺	☺	☺	☺	☺	☺	☺
Nevler, 2020 [24]	?	?	☺	?	☺	☺	☺
Norel, 2018 [29]	?	?	☺	☺	☺	☺	☺
Peplinski, 2019 [25]	☺	☺	☺	☺	☺	☺	☺
Robert, 1999 [39]	☺	☺	?	☺	☺	☺	☺
Rong, 2015 [16]	☺	☺	☺	☺	☺	☺	☺
Rong, 2016 [40]	☺	☺	☺	☺	☺	☺	☺
Rong, 2020 [19]	☺	?	?	?	☺	☺	☺
Rowe, 2022 [20]	?	?	☺	?	☺	☺	☺
Rutkove, 2020 [21]	?	☺	☺	☺	☺	☺	☺
Silbergleit, 1997 [41]	?	☺	☺	☺	☺	☺	☺

Stegmann, 2020 [26]	?	☺	☺	☺	☺	☺	☺
Tanchip, 2022 [42]	?	?	☺	?	☺	☺	☺
Tena, 2022 [43]	?	?	?	?	☺	☺	☺
Tomik, 2015 [44]	?	?	☺	?	☺	☺	☺
Tomik, 1999 [45]	☺	☺	☺	☺	☺	☺	☺
Vashkevich, 2018 [61]	?	☺	☺	☺	☺	☺	☺
Vashkevich, 2019 [60]	?	☺	☺	☺	☺	☺	☺
Vashkevich, 2021 [59]	?	☺	☺	☺	☺	☺	☺
Wang, 2018 [46]	?	?	☺	☺	☺	☺	☺
Wang, 2016 [47]	?	?	☺	☺	☺	☺	☺
Wang, 2016 [48]	?	☺	☺	☺	☺	☺	☺
Weismer, 2001 [49]	?	☺	?	☺	☺	☺	☺
Wisler, 2019 [50]	?	?	☺	☺	☺	☺	☺
Yunusova, 2016 [9]	☺	☺	☺	☺	☺	☺	☺

Green square= Low risk of bias. Orange square= High risk of bias. Blue square= Unclear risk of bias
